# Adductor Canal Block Versus Femoral Nerve Block for Postoperative Pain Management in Anterior Cruciate Ligament Reconstruction: A Prospective Interventional Study

**DOI:** 10.7759/cureus.64625

**Published:** 2024-07-15

**Authors:** Tapan Dhumey, Nikhil Bhalerao, Amreesh Paul, Dnyanshree Wanjari

**Affiliations:** 1 Anaesthesiology, Jawaharlal Nehru Medical College, Datta Meghe Institute of Higher Education and Research, Wardha, IND

**Keywords:** visual analog scale, regional anesthesia, quadriceps muscle strength, bupivacaine, analgesia, postoperative pain management, femoral nerve block, adductor canal block, anterior cruciate ligament reconstruction

## Abstract

Background

A common knee joint disorder is injury to the anterior cruciate ligament (ACL), which often requires surgery. Proper pain control after the surgery facilitates fast recovery and prevents chronic pain. To provide analgesia for knee procedures, the use of opioids, non-steroidal anti-inflammatory medications, and regional techniques are commonly employed. This study aims to evaluate the efficacy of adductor canal block (ACB) and femoral nerve block (FNB) for postoperative pain management after anterior cruciate ligament reconstructions (ACLRs).

Methodology

This prospective interventional study included 30 participants scheduled for patellar graft ACLR. They were assigned into groups, i.e., ACB and FNB, with 15 patients each. The evaluation occurred one day before the operation, and all surgical procedures were performed using spinal anesthesia. During the postoperative period, a 10-point visual analog scale (VAS) was utilized to quantify pain intensity at the end of the surgery and at various intervals after the surgery. Patients with a VAS score greater than 4 received either FNB or ACB using bupivacaine 0.125%. Duration of analgesia time, power of quadriceps muscle, and neurologic complications were documented.

Results

No statistically significant value was observed in the mean duration of analgesia between the patients in ACB (348.33 minutes) and the patients in FNB (363.06 minutes). No motor block was observed in 12 patients who received ACB, while only four patients had a motor-sparing effect among those who received FNB. No neurological adverse effects were observed in the study participants.

Conclusions

ACB provides an equal duration of analgesia similar to FNB, and ACB significantly spares motor strength and maintains higher quadriceps power than FNB.

## Introduction

One of the most often treated disorders involving the knee is the anterior cruciate ligament (ACL) injury. It is a frequently encountered injury in athletes [1]. Pain is an unpleasant feeling that results from ongoing or potential tissue injury [2]. A prompt restoration to normal physiological function is facilitated by adequate pain management, inhibiting the onset of chronic pain. Adequate analgesia is, therefore, crucial for recovery from anterior cruciate ligament reconstruction (ACLR) surgery. An appropriate pain management plan is necessary for a speedy discharge because the frequency of ACLR is increasing, and most of these procedures are performed in outpatient settings because of their productive and economical results [3,4]. In particular, peripheral nerve blocks used for regional anesthesia are essential for maximizing postoperative pain management and facilitating discharge. However, the best strategy differs [5]. Opioids, non-steroidal anti-inflammatory drugs (NSAIDs), and regional techniques are the mainstays of traditional analgesia during the recovery phase [6].

In comparing systemic opioids with femoral nerve block (FNB), the latter has been proven to offer significant and additional pain relief and can decrease the duration of hospitalization after knee surgery. Therefore, it is administered with neuraxial or general anesthesia to ensure adequate analgesia after ACLR [7,8]. FNB is associated with significant quadriceps muscle weakness and an increased propensity for falls and is observed during the postoperative period for up to six weeks, even though the same method is efficient in pain relief and minimizing the use of analgesics [9]. Another wheelchair-sparing technique, targeting primarily the saphenous nerve and the nerve to vastus medialis, is the adductor canal block (ACB) [10]. It is a promising, similar, effective pain relief technique for ACLR compared to FNB but without quadriceps muscle loss [11]. Therefore, administering ACB allows for earlier discharge and improved patient comfort by controlling postoperative pain and maintaining the strength of the quadriceps muscle [12].

This study compares the efficacy of ACB and FNB in managing pain following ACL surgery. The primary objective is to evaluate and contrast the postoperative analgesic duration offered by ACB and FNB. The secondary objectives are comparing the quadriceps strength following block administration and evaluating any adverse effects, such as postoperative neurological symptoms.

## Materials and methods

Study design and inclusion and exclusion criteria

**Figure 1:**
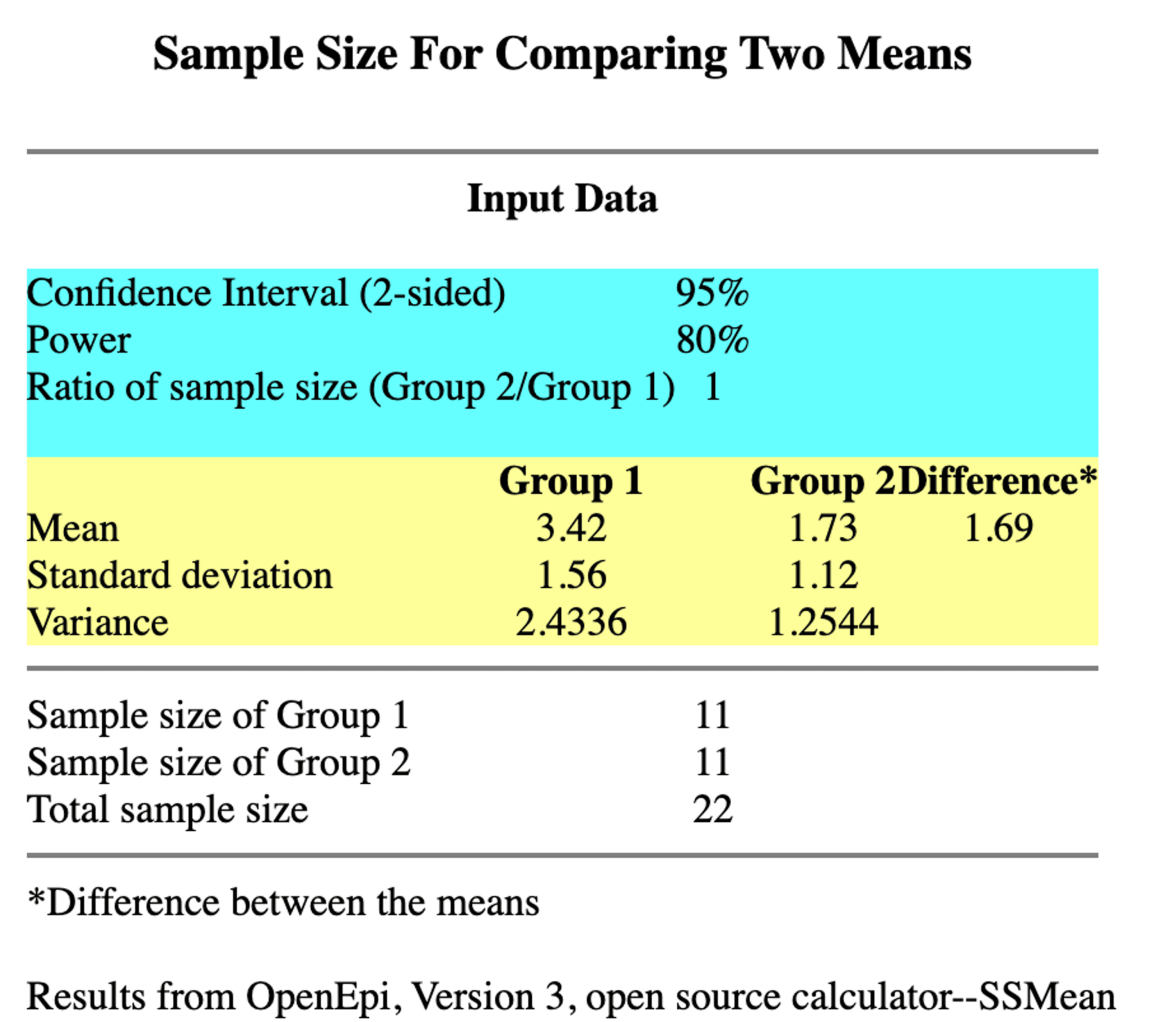
Sample size calculation. Based on the study by Ghodki et al. [13].

This prospective interventional study was conducted at Jawaharlal Nehru Medical College (JNMC), Datta Meghe Institute of Higher Education and Research (DMIHER), Sawangi, Wardha, following approval from the institutional ethical committee. The sample size was calculated using openepi.com (Figure 1) by comparing the numeric rating scale scores between the two groups in the study by Ghodki et al. [13]. A sample of 11 patients was required in both groups to have statistical significance. Hence, the study was planned to be conducted among 30 patients, with 15 patients in each group, to account for possible dropouts during the study. The study included 30 patients who were scheduled for graft ACLR. Patients belonging to any gender, aged between 18 and 45 years, belonging to American Society of Anesthesiologists (ASA) class I and II, height between 150 and 180 cm, and weight between 40 and 70 kgs were included. Non-cooperative patients, uncontrolled diabetics and hypertensives, lactating or pregnant females, patients known to have allergic reactions to the drugs, and those not providing valid consent were excluded.

Preoperative evaluation

As part of the preoperative preparation, one day before the surgery, each patient underwent a pre-anesthetic evaluation. This involved taking a complete medical history, performing a systemic general examination, and conducting relevant diagnostic tests such as blood coagulation profile, serum electrolytes, liver and renal function test, random blood sugar, and complete blood count.

Data collection method

Two groups of 30 patients were randomly assigned using a computer-generated randomization table (group FNB and group ACB). During the procedure, an 18-gauge cannula was used to establish intravenous access, and 500 mL of Ringer’s lactate infusion was given at a rate of 10 mL/kg for 30 minutes, following maintenance dosages. Vital monitoring was performed using standard ASA monitoring devices, including an electrocardiogram, non-invasive blood pressure, and pulse oximeter. Using a 25-gauge Quincke needle and 15 mg of bupivacaine hydrochloride, hyperbaric, spinal anesthesia was administered at the L3-L4 level to reach a sufficient T12 spinal level. The surgery was conducted following this. Following the surgery, measurements were made of the patient’s oxygen saturation, mean arterial pressure, and heart rate. If the results were within normal ranges, they were moved to the recovery area.

According to their group assignment, the participants received ultrasound-guided ACB or FNB after surgery with 15 mL of 0.125% bupivacaine under aseptic circumstances according to the study by Ghodki et al. [13]. Using a 10-point visual analog scale (VAS), the intensity of pain was recorded during the postoperative period at different times (0, 2, 4, 6, 8, 10, 12, and 24 hours after the operation) [14]. When patients’ VAS score was greater than 4 after receiving a block, rescue analgesia of 75 mg of diclofenac was given intramuscularly.

The amount of time from when a block was administered until the first rescue analgesic was needed (VAS score >4) was considered the duration of analgesia. The participants performed a straight leg raise to measure their quadriceps muscular power when assessing motor blockage. Up to eight hours following surgery, the motor blockage was rated every two hours using the modified Bromage scale [15]. Throughout the trial, the incidence of postoperative neurological symptoms, such as prolonged paraesthesia or numbness, weakness, or non-surgical pain in the operated extremity, as well as nausea and vomiting were noted.

Statistical analysis

For continuous variables, including age, the duration of analgesia, VAS scores, and quadriceps muscle strength, descriptive statistics were computed. For categorical variables, including gender, the extent of motor block, and neurological problems, frequencies and percentages were utilized. Before performing t-tests, a normality test was conducted to determine if the data followed a normal distribution, which is essential for the validity of the t-test. The Shapiro-Wilk test was employed for this purpose, where the null hypothesis is that the data follows a normal distribution. t-tests were used to compare baseline features, while chi-square tests were used to compare categorical variables. The VAS data were analyzed using analysis of variance. Using t-tests, the duration of analgesia in each group was compared. t-tests were used to compare the quadriceps muscle strength in both groups, and chi-square tests were used to investigate the modified Bromage scale values. Furthermore, the incidence of neurological complications and motor block were compared using chi-square tests. SPSS version 20 (IBM Corp., Armonk, NY, USA) was employed to analyze the data. P-values less than 0.05 were considered statistically significant. Figures and tables were used to illustrate the data findings.

## Results

Patients’ demographics and surgical times were similar in the two groups (Table 1).

**Table 1:** The demographic data of the patients. ACB = adductor canal block; ASA = American Society of Anesthesiologists; BMI = body mass index; FNB = femoral nerve block

	FNB	ACB	P-value
Age in years, mean (SD)	27.80 (4.42)	27.66 (3.28)	1.00
Gender (male/female)	9/6	9/6	1.00
ASA (I/II)	12/3	15/0	0.22
BMI (kg/m^2^), mean (SD)	23.60 (3.71)	24.66 (4.38)	0.47
Duration of surgery (minutes), mean (SD)	165.33 (5.32)	160.13 (7.46)	0.037

The duration of analgesia offered by either block did not differ significantly (Table 2). The duration of the blocks’ analgesic effects was similar in both groups. The mean duration of analgesia in the ACB and FNB groups was 348.33 and 363.06 minutes, respectively. The duration of analgesia did not differ statistically significantly (p = 0.12).

**Table 2:** Duration of analgesia in minutes. ACB = adductor canal block; FNB = femoral nerve block; NS = not significant

	Participants	Mean	Standard deviation	Standard error mean	P-value
ACB	15	348.33	26.70	6.89	0.12, NS
FNB	15	363.06	24.09	6.22	

After the block, the quadriceps muscle’s motor power was assessed. The motor power in the ACB group was much higher than the FNB group (Table 3). Patients who underwent an ACB exhibited notably higher motor power in their quadriceps muscle following the block than those who underwent an FNB. Only 26.67% of patients in the FNB group attained the Grade 0 Bromage score, which indicates full leg and foot movement, compared to most patients in the ACB group (80%). At a p-value of 0.029, this difference was statistically significant.

**Table 3:** Bromage scale grading. ACB = adductor canal block; FNB = femoral nerve block; S = significant

Bromage score	ACB group	FNB group	P-value
Grade 0	12 (80%)	4(26.67%)	0.029, S
Grade 1	2 (13%)	5(33.33%)	
Grade 2	1 (6.67%)	5 (33.33%)	
Grade 3	0 (0%)	1 (6.67%)	
Grade 4	0 (0%)	0 (0%)	
Total	15 (100%)	15 (100%)	

During the course of the study, neither group experienced difficulties. This included the absence of nausea or vomiting during and postoperative neurological problems such as persistent numbness or paraesthesia, weakness, or non-surgical pain in the operated extremities.

## Discussion

The ACL is the most frequent knee ligament to tear, and most often requires surgical correction [16]. As spinal anesthesia is safe, dependable, produces quick neuraxial blockade, and is relatively simple to administer, it is the most often used regional anesthetic technique [17]. The primary drawbacks of spinal anesthesia are its brief duration of action and the fact that, when used exclusively with local anesthetics, it does not offer extended postoperative analgesia [18]. Pain is generally considered a significant post-surgical consequence that, if ignored, can have major adverse impacts. Managing acute postoperative pain is a challenging and important issue [19]. With the advent of technologies such as peripheral nerve stimulators and ultrasonography, peripheral nerve blocks are becoming more and more common during infraumbilical procedures. They offer prolonged postoperative analgesia along with stable hemodynamics [20]. Research indicates that ACB effectively delivers sufficient pain relief following knee replacement surgery. Many studies compare ACB with FNB in knee arthroscopic procedures, and the majority of them focus on the motor-sparing effects and level of analgesia that either block provides. We decided to limit our investigation to ACLR and incorporate the measurement of quadriceps muscle power and the length of analgesia supplied by blocks into the criteria under investigation.

Duration of analgesia

Our research revealed no discernible variation in the duration of analgesia brought on by FNB and ACB. Our results align with the study conducted by Ghodki et al. comparing ACB guided by ultrasonography with FNB for arthroscopic ACL replacement under general anesthesia [13]. Our results also coincide with the study by Abdallah et al. [21]. The timing of the first postoperative analgesic request and analgesic requirements were comparable between the two research groups as the cited study.

In the study by Kim et al., ACBs were comparatively more effective than FNBs in terms of opioid intake at 24 and 48 hours after anesthesia or numeric rating scale pain levels [22]. However, El Ahl compared ACB and FNB for postoperative pain control ACLR using ropivacaine [23]. In the study, patients in the ACB group consumed statistically more rescue analgesia overall and had high VAS scores at 18 and 24 hours compared to the FNB group.

Assessment of quadriceps strength

More so than FNB, we discovered that ACB maintains the quadriceps muscle strength. In a prior study, Jaeger et al. compared the effects of ACB against FNB on quadriceps strength in young, healthy volunteers [24]. They found that the decline in the motor power of the quadriceps muscle from baseline value was 49% with FNB compared to 8% with ACB. Our findings also align with the research conducted by Koh et al., which discovered that ACB, as opposed to FNB, spares quadriceps strength, facilitating early mobilization [25]. Abdallah et al. and El Ahl et al. discovered comparable findings, indicating that ACB sustains more quadriceps power than FNB [21,23]. The femoral nerve targeted by the FNB contains motor nerve fibers, likely contributing to the observed differences in muscle strength between the ACB and FNB groups. This is an important consideration as motor nerve involvement can lead to reduced quadriceps strength in patients receiving FNB. The reduction in quadriceps strength associated with FNB may delay rehabilitation progress and extend the time required for patients to return to their pre-injury levels of physical activity and sports participation. Athletes, in particular, rely heavily on quadriceps strength for explosive movements, stability, and overall performance. A delay in regaining full quadriceps strength could potentially reduce the rate of return to sport and extend the overall recovery period.

Complications after block administration

Following block administration, patients in both groups were assessed for the development of neurological sequelae such as non-surgical pain, weakness, or prolonged numbness or paraesthesia in the operative extremity. None of the patients were observed to have any of these complications. Additionally, during the trial, no group reported any cases of local anesthetic systemic toxicity. Our findings align with the research conducted by Kim et al., Wang et al., and El Ahl et al. [22,23,26].

The choice between ACB and FNB should be guided by clinical considerations such as the desired duration of analgesia and the importance of preserving quadriceps strength. ACB offers comparable analgesic efficacy to FNB while potentially minimizing motor blockade and facilitating earlier mobilization. This advantage may be particularly beneficial in patients requiring rapid recovery, such as athletes and active individuals.

Limitations

Limitations of the study as a single-center trial could introduce bias, and the short-term follow-up period of up to 24 hours post-surgery might not capture longer-term outcomes or complications that could arise beyond this timeframe. Another potential limitation is the use of a numeric rating scale (VAS) for pain assessment, which is subjective and may vary between individuals. Lastly, while efforts were made to minimize biases through randomization and blinding, the possibility of unmeasured confounders influencing outcomes cannot be completely ruled out.

## Conclusions

It has been shown in this prospective study that, for patients who have had ACL repair surgery, both FNB and ACB can effectively provide postoperative analgesia. Both FNB and ACB had postoperative analgesia for the same duration. Patients receiving either type of block reported comparable pain relief in both groups. Compared to FNB, ACB significantly preserved quadriceps muscular strength. The motor function was better in the ACB group for early mobilization and rehabilitation purposes. This is crucial for athletes and others wanting to return to their activities as soon as possible. Both blocks had minimal complications. During the study, no significant incidences of local anesthetic toxicity or neurological side effects such as numbness, paraesthesia, or weakness were experienced by any of the patients. Hence, the safety profile of both blocks was found to be good, with both being excellent options for knee surgery pain relief.

## References

[1] Rodriguez K, Soni M, Joshi PK (2021). Anterior cruciate ligament injury: conservative versus surgical treatment. Cureus.

[2] Paul A, Borkar A (2022). Fluoroscopy-guided splanchnic nerve block for cancer-associated pain. Cureus.

[3] Paul A, Borkar A, Bhalerao N, Wanjari D (2023). A Comparative evaluation of intra-articular bupivacaine vs bupivacaine and dexmedetomidine for postoperative analgesia in arthroscopic knee surgeries. Cureus.

[4] Wang X, Jia D, Chen X, Xu Y (2013). Comparison of intra-articular low-dose sufentanil, ropivacaine, and combined sufentanil and ropivacaine on post-operative analgesia of isolated anterior cruciate ligament reconstruction. Knee Surg Sports Traumatol Arthrosc.

[5] Kamel I, Ahmed MF, Sethi A (2022). Regional anesthesia for orthopedic procedures: what orthopedic surgeons need to know. World J Orthop.

[6] Schwenk ES, Mariano ER (2018). Designing the ideal perioperative pain management plan starts with multimodal analgesia. Korean J Anesthesiol.

[7] Rodriguez-Patarroyo FA, Cuello N, Molloy R, Krebs V, Turan A, Piuzzi NS (2021). A guide to regional analgesia for total knee arthroplasty. EFORT Open Rev.

[8] Hasabo EA, Assar A, Mahmoud MM (2022). Adductor canal block versus femoral nerve block for pain control after total knee arthroplasty: a systematic review and meta-analysis. Medicine (Baltimore).

[9] Runner RP, Boden SA, Godfrey WS, Premkumar A, Samady H, Gottschalk MB, Xerogeanes JW (2018). Quadriceps strength deficits after a femoral nerve block versus adductor canal block for anterior cruciate ligament reconstruction: a prospective, single-blinded, randomized trial. Orthop J Sports Med.

[10] Rasouli MR, Viscusi ER (2017). Adductor canal block for knee surgeries: an emerging analgesic technique. Arch Bone Jt Surg.

[11] Tan M, Chen B, Li Q, Wang S, Chen D, Zhao M, Cao J (2024). Comparison of analgesic effects of continuous femoral nerve block, femoral triangle block, and adductor block after total knee arthroplasty: a randomized clinical trial. Clin J Pain.

[12] Fan Chiang YH, Wang MT, Chan SM (2023). Motor-sparing effect of adductor canal block for knee analgesia: an updated review and a subgroup analysis of randomized controlled trials based on a corrected classification system. Healthcare (Basel).

[13] Ghodki PS, Shalu PS, Sardesai SP (2018). Ultrasound-guided adductor canal block versus femoral nerve block for arthroscopic anterior cruciate ligament repair under general anesthesia. J Anaesthesiol Clin Pharmacol.

[14] Klimek L, Bergmann KC, Biedermann T (2017). Visual analogue scales (VAS): measuring instruments for the documentation of symptoms and therapy monitoring in cases of allergic rhinitis in everyday health care: Position Paper of the German Society of Allergology (AeDA) and the German Society of Allergy and Clinical Immunology (DGAKI), ENT Section, in collaboration with the working group on Clinical Immunology, Allergology and Environmental Medicine of the German Society of Otorhinolaryngology, Head and Neck Surgery (DGHNOKHC). Allergo J Int.

[15] Jaremko I, Lukaševič K, Tarasevičius Š, Zeniauskas L, Macas A, Gelmanas A (2021). Comparison of 2 peripheral nerve blocks techniques for functional recovery and postoperative pain management after total knee arthroplasty: a prospective, double-blinded, randomized trial. Med Sci Monit.

[16] Shom P, Varma AR, Prasad R (2023). The anterior cruciate ligament: principles of treatment. Cureus.

[17] Poots C, Chin KJ (2024). Strategies for successful lumbar neuraxial anaesthesia and analgesia in patients with challenging anatomy. BJA Educ.

[18] Gürkan Y, Canatay H, Ozdamar D, Solak M, Toker K (2004). Spinal anesthesia for arthroscopic knee surgery. Acta Anaesthesiol Scand.

[19] Francis AP, Modak A (2021). A comparative evaluation of intra-articular bupivacaine vs bupivacaine and dexmedetomidine for postoperative analgesia in arthroscopic knee surgeries: a study protocol. J Pharm Res Int.

[20] Bansal L, Attri JP, Verma P (2016). Lower limb surgeries under combined femoral and sciatic nerve block. Anesth Essays Res.

[21] Abdallah FW, Whelan DB, Chan VW (2016). Adductor canal block provides noninferior analgesia and superior quadriceps strength compared with femoral nerve block in anterior cruciate ligament reconstruction. Anesthesiology.

[22] Kim DH, Lin Y, Goytizolo EA (2014). Adductor canal block versus femoral nerve block for total knee arthroplasty: a prospective, randomized, controlled trial. Anesthesiology.

[23] El Ahl MS (2015). Femoral nerve block versus adductor canal block for postoperative pain control after anterior cruciate ligament reconstruction: a randomized controlled double blind study. Saudi J Anaesth.

[24] Jaeger P, Nielsen ZJ, Henningsen MH, Hilsted KL, Mathiesen O, Dahl JB (2013). Adductor canal block versus femoral nerve block and quadriceps strength: a randomized, double-blind, placebo-controlled, crossover study in healthy volunteers. Anesthesiology.

[25] Koh IJ, Choi YJ, Kim MS, Koh HJ, Kang MS, In Y (2017). Femoral nerve block versus adductor canal block for analgesia after total knee arthroplasty. Knee Surg Relat Res.

[26] Wang D, Yang Y, Li Q (2017). Adductor canal block versus femoral nerve block for total knee arthroplasty: a meta-analysis of randomized controlled trials. Sci Rep.

